# Cephalometric landmark detection without X-rays combining coordinate regression and heatmap regression

**DOI:** 10.1038/s41598-023-46919-x

**Published:** 2023-11-16

**Authors:** Kaisei Takahashi, Yui Shimamura, Chie Tachiki, Yasushi Nishii, Masafumi Hagiwara

**Affiliations:** 1 Department of Information and Computer Science, Faculty of Science and Technology, Keio University, Kanagawa, 223-8522 Japan; 2 Department of Orthodontics, Tokyo Dental College, Tokyo, 101-0061 Japan

**Keywords:** Computer science, Information technology, Orthodontics

## Abstract

Fully automated techniques using convolutional neural networks for cephalometric landmark detection have recently advanced. However, all existing studies have adopted X-rays. The problem of direct exposure of patients to X-ray radiation remains unsolved. We propose a model for detecting cephalometric landmarks using only facial profile images without X-rays. First, the model estimates the landmark coordinates using the features of facial profile images through high-resolution representation learning. Second, considering the spatial relationship of the landmarks, the model refines the estimated coordinates. The estimated coordinates are input into fully connected networks to improve the accuracy. During the experiment, a total of 2000 facial profile images collected from 2000 female patients were used. Experiments results suggested that the proposed method may perform at a level equal to or potentially better than existing methods using cephalograms. We obtained an MRE of 0.61 mm for the test data and a mean detection rate of 98.20% within 2 mm. Our proposed two-stage learning method enables a highly accurate estimation of the landmark positions using only facial profile images. The results indicate that X-rays may not be required when detecting cephalometric landmarks.

## Introduction

Quantitative maxillo-facial morphology evaluation is one of the essential steps in orthodontic treatment. In particular, a cephalometric analysis^1^ is crucial to evaluate dentofacial proportions, clarify the anatomic basis for malocclusion and establish orthodontic treatment planning. Moreover, that can recognize and evaluate changes brought about by orthodontic treatment. Although dedicated software is usually employed for such an analysis, tracing the maxillo-facial structure and pointing out the landmarks on the lateral cephalograms must be conducted manually by orthodontic specialists. However, these manual procedures are time-consuming and lead to intra- and inter-person variations^2^. Furthermore, the wrong diagnosis caused by inaccurate tracing should induce serious treatment results.

Automated cephalometric analysis systems have recently been developed^2–20^. Previous studies have adopted knowledge bases^5^ and pattern matching^3,4^ for landmark detection. However, the detection accuracies of these studies are not clinically acceptable^6^. Recently developed algorithms can be divided into two categories: random forest and convolutional neural networks (CNNs). The IEEE International Symposium on Biomedical Imaging (ISBI) , held in 2014 and 2015^12,13^ , posed the task of detecting 19 landmarks from lateral cephalograms. At ISBI, most of the studies^14,16^ used classical machine learning focused on a random forest. A random forest is usually complex and vulnerable to an overfitting^21^. In the past few years, deep learning methods for landmark detection have outperformed methods using a random forest. In particular, CNN-based methods^2,6–11,17–20^ have achieved remarkable results. A CNN is a deep machine learning technique inspired by visual biological recognition, and has been demonstrated to be effective in cephalometric landmark detection^17^. CNN-based methods are often implemented in several stages^2,6–9,11,18–20^. In the first stage, candidate landmarks are identified by searching for local patterns in the cephalograms. In the next stage, the landmarks are finetuned to improve the accuracy. This approach suffers from a performance gap^19^ between coordinate-^6,7,10,17,18,20^ and heatmap-^2,8,9,11,19^, based methods. Coordinate regression methods adopt a regression model to directly predict the *x*- and *y*-coordinates of the landmarks, and it can be expected to make predictions that incorporate the structural knowledge of the landmarks; however they are not as accurate as a heatmap regression method. By contrast, the heatmap regression methods formulate landmark detection as a regression problem that estimates a heatmap of the landmark locations. Although they have achieved high accuracy, having the ability to exploit local features of the images, they have difficulty incorporating structural knowledge among the different landmarks. In those studies, several challenges remain, such as vulnerability to image distortions like occlusion and the difficulty of detecting certain cephalometric landmarks^7,8,13,19^. Therefore, a few studies^9,10,19^ have proposed training methods that consider the local and anatomical features simultaneously. However, a significant enhancement in accuracy has yet to be achieved, and efforts to solve this problem have recently commenced. Furthermore, there have been recent attempts to investigate the potential of cephalometric landmark detection by applying originally created datasets to deep learning models^11,20^.

As these previous studies were based on the assumption that cephalograms would be used, X-ray exposure is inevitable. As the exposure dose of cephalography is $2-3$ µSv, cone beam computed tomography (CBCT) is $20-850$ µSv for the maxillofacial procedures^22^. Therefore, these doses would be below the limits. Nevertheless, the risk of X-ray exposure to patients, especially children and pregnant women, in dental practice still exists. There is a demand for research on alternative techniques requiring no or low exposure to X-rays^22^.

Facial landmark detection localizes predefined facial landmarks such as the eyes, nose, mouth, and chin from facial images. Previous studies have adopted the active shape model (ASM)^23^ or constrained local model (CLM)^24^ for detecting landmarks under certain restrictions. However, the robustness of these studies needs to be enhanced against various changes in appearance. A cascade regression approach^25^ was studied to address this problem. However, a cascade regression is limited in deepening the structure for increased accuracy^26^. Deep neural networks (DNN) have recently been adopted as a powerful alternative^27^. In addition, CNN-based approaches have exhibited remarkable results. In particular, models with an hourglass structure^28^ and heat-map-based regression^29–31^ achieve high accuracy. Sun et al.^31^ proposed HRNetV2, which uses a high-resolution network (HRNet)^32^ for learning high-resolution representations, with the hourglass structure^28^ being the mainstream. The structure of HRNetV2 connected high- to low-resolution convolutions in parallel, and it was possible to maintain a high-resolution throughout. Consequently, the performance of HRNetV2 was equal to or greater than that of conventional methods, while reducing the number of parameters and the computational cost. There is also study^33,34^ focused on learning algorithms that do not depend on model structure. In fact, ADNet^29^ is based on LAB^33^ and Awing^34^ and has shown high performance on many datasets.

In this paper, we present a novel cephalometric landmark detection method that incorporates a highly accurate facial landmark detection model. The proposed method is trained without cephalograms. We adopt HRNetV2^31^, which achieves a high accuracy in a wide range of visual tasks by generating a high-resolution representation with accurate spatial information. In addition, we combine the heatmap regression model with a coordinate regression model. This solves the problem using conventional heatmap regression models, which incurs difficulty in learning anatomical features between landmarks. The proposed method comprises two stages: cephalometric landmark localization using HRNetV2 and refinement of landmark positions using multilayer perceptron (MLP). MLP contributes to the estimation reflecting the spatial relationship between landmarks. The inputs of the model are not cephalograms, as in the past, but facial profile images. To the best of our knowledge, this is the first study on cephalometric landmark detection without the use of cephalograms. During the experiment, we used facial profile images provided by the Tokyo Dental College. Two clinical orthodontists (with 3 and 15 years of experience, respectively) plotted a total of 23 landmarks on each of 2000 images based on the cephalograms, and one clinical orthodontist (with 36 years of experience) reviewed the results. The proposed method achieves a mean radial error (MRE) significantly below the clinically acceptable error of 2.0 mm^6^. Specifically, we obtained an MRE of 0.61 mm for the test data and a mean detection rate 98.20% for the threshold. While a direct comparison is difficult, we have suggested the possibility of achieving performance equal to or potentially better than existing methods using cephalograms. The main contributions of the proposed method are presented as follows:

- We proposed a cephalometric landmark detection without the use of cephalograms, and demonstrated performance at a level that is considered clinically acceptable.
- We achieved a significant improvement in accuracy by combining the CNN-based method with a conventional MLP.

## Materials and methods

The study protocol was approved by the Institutional Review Board (IRB) of our institutes (Tokyo Dental College, No. 1091, 2021-12-17). All methods were carried out in accordance with the Helsinki Declaration principles and relevant guidelines and regulations. Informed consent was obtained from all the patients, and written informed consent was obtained from three patients, including the facial images, allowing us to use their information/images in an online open-access publication.

**Figure 1:**
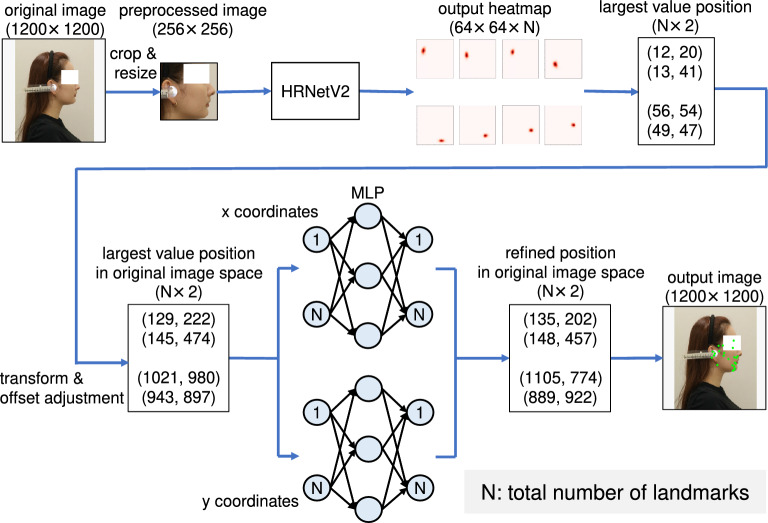
Overview of the proposed model. The model is divided into HRNetV2 and MLP. After HRNetV2 applies the heatmap regression based on the feature extraction, MLP estimates the coordinates based on the spatial relationship of the landmarks.

Figure 1 presents an overview of the proposed method. The proposed method comprises two stages: HRNetV2^31^ and MLP. Unlike with existing studies, we used the facial profile images as input instead of a cephalogram. First, all landmark locations in the input images are estimated through heatmap regression using HRNetV2. In this step, the model learns the relationship between the local features of the image and landmarks. As described later in *Ablation Study* section, the accuracy of the estimation is insufficient, however. Therefore, we introduced a coordinate regression by MLP, which significantly improved the accuracy of landmark estimation. MLP was adopted to estimate the spatial location of the landmarks, i.e., their relative location. This two-stage approach, coarse estimation using heatmap regression and a fine estimation using MLP, enables the accurate detection of all landmarks.

**Figure 2:**
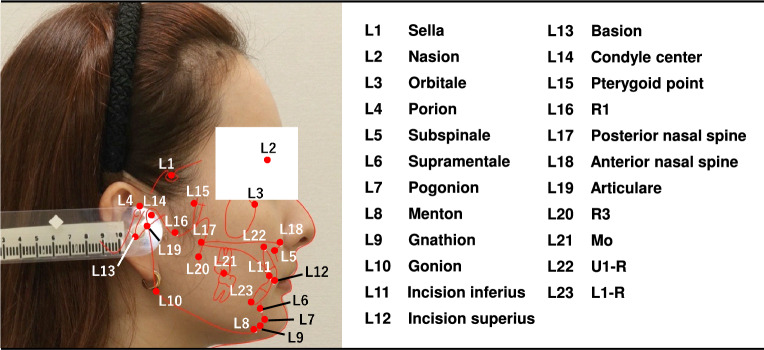
Illustration of 23 cephalometric landmarks.

**Figure 3:**
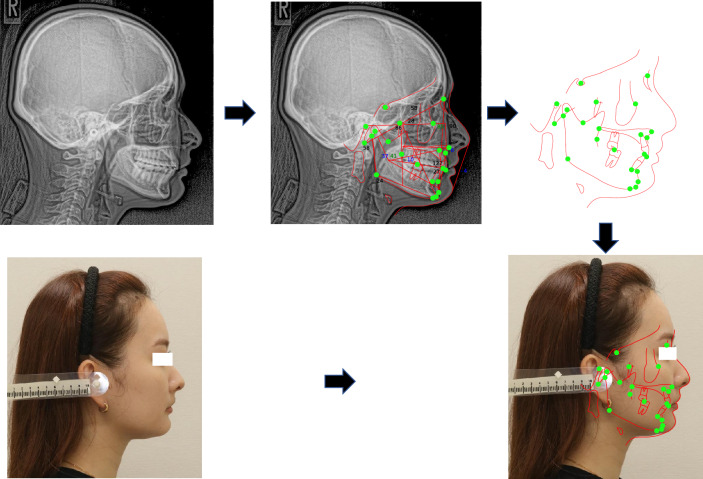
Procedure for plotting landmarks and superimposing the cephalometric tracing on the facial profile image.

**Figure 4:**
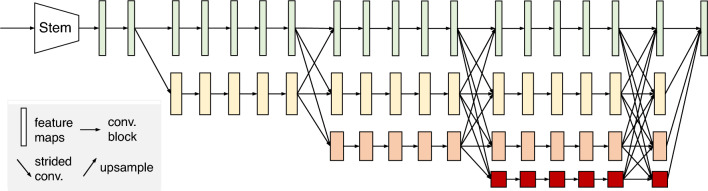
HRNetV2 structure^31^. HRNetV2 comprises four stages connected in parallel by high- to low-resolution subnetworks. The horizontal and vertical sizes of the feature maps correspond to the resolution and number of channels, respectively.

**Figure 5:**
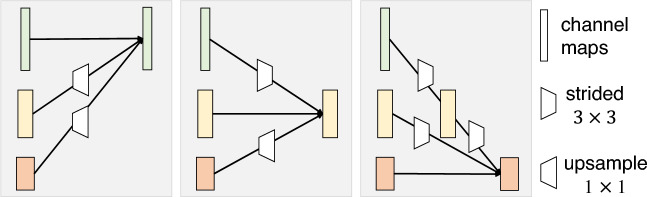
Illustration of how to aggregate information of multiple resolutions using an exchange unit^32^. A stride $3\times 3$ convolution is adopted for downsampling. Nearest neighbor sampling followed by a $1\times 1$ convolution is used for upsampling.

### Dataset

A total of 2000 lateral cephalograms and profile photograph images collected from 2000 female patients aged 14 to 69 years (mean age of 27 years) were provided by the Tokyo Dental College. These images were acquired in jpeg format using a CX-150S (ASAHIROENTGEN, Tokyo, Japan) and an EOS Kiss X90 (Canon, Tokyo, Japan). The standard for taking lateral cephalogram is set worldwide. The film should be kept parallel to the mid-sagittal plane of the head and set the head with ear rods so that the center line of the X-ray beam passes through the axes of the left and right ear rods. The distances from the X-ray tube to the mid-sagittal plane of the head and from the mid-sagittal plane of the head to the film is assumed to be 150 cm and 15 cm, respectively. The method of taking a profile photograph image is the same as that of a lateral cephalogram. That is, the distance from the camera to the midsagittal plane of the head is 150 cm, and the head is fixed with ear rods. The skeletal information obtained from the lateral cephalograms was superimposed on the profile photograph images using Quick Ceph Studio (ver. 5.0.2, Quick Ceph Systems, San Diego, California). The 23 landmarks illustrated in Fig. 2 were manually plotted by two orthodontists and finally checked by an experienced orthodontist. The two of them are the board members of Orthodontic specialists. These 23 landmarks were selected from the measurement points of the Downs method, Northwestern method, and a Ricketts analysis. Coordinates of landmarks on the lateral image were identified by imglab, an annotation tool included with Dlib^35^. Each image has $1200\times 1200$ pixels and the pixel spacing for the device was 0.35. We present the averaged results of the experiment based on fivefold cross-validation, each containing 400 test and 1600 training profile photograph images, respectively.

### Superimposition of the cephalometric tracing on the facial profile image

Figure 3 shows the procedure for plotting landmarks and superimposing the cephalometric tracing on the facial profile image conducted as follows:

1. Plot 23 landmarks and trace the profile (from forehead to upper lip) on the cephalogram within the computer screen of Orthodontic analysis software (Quick Ceph Studio).
2. Separate the set of the landmark plots and the cephalometric tracing on the screen.
3. Superimpose the cephalometric tracing on the facial profile image by manually matching the line from nose to upper lip of the tracing with that of the facial profile image on another screen.

Five sets of the landmark plots and the cephalometric tracing created in step 1 were randomly selected and the same set was superimposed on the facial profile image by each observer according to steps 2 and 3. We measured the intra- and inter-observer landmark distance errors for five landmarks (Sella, Porion, Menton, Gonion, Basion) to confirm the accuracy and reliability of the superimposition. The reason of selecting the five landmarks, it can be considered that the further away from the profile tracing line the larger the error because the superimposition is conducted based on the profile tracing line as shown in step 3. Therefore, the five points farthest from the profile tracing line were selected. To evaluate intra-observer variability, superimpositions were conducted by one orthodontist three times with an interval of two weeks. To evaluate inter-observer variability, superimpositions were conducted by three orthodontists. The two of them are the board member of Orthodontic specialist. The mean and standard deviation of the intra- and inter-observer landmark distance error and the intraclass correlation coefficient (ICC) for the landmark plots were calculated for each set with 95% confidence intervals. In calculating the ICC, the Shapiro-Wilk test was used to determine whether the data were normally distributed. All statistical evaluations were conducted by SPSS software (ver. 27.0, IBM, Armok, New York).

### Repeatability and reproducibility test of manual landmark plotting

To compare the repeatability and reproducibility of orthodontists’ landmark prediction errors, a total of five cephalograms from five patients were randomly selected and 23 landmarks were plotted. To evaluate intra-observer variability, cephalometric landmark plotting was conducted by one orthodontist three times with an interval of two weeks. To evaluate inter-observer variability, cephalometric landmark plotting was conducted by three orthodontists. The mean and standard deviation of the intra- and inter-observer prediction error and the ICC for each landmark were calculated for each set with 95% confidence intervals. In calculating the ICC, the Shapiro-Wilk test was used to determine whether the data were normally distributed. All statistical evaluations were conducted by SPSS software.

### Heatmap regression

To estimate the location of landmarks, we used HRNetV2 to generate heatmaps of the landmarks. Figure 4 shows the structure of HRNetV2^31^, which starts with a high-resolution subnetwork (stem) and adds high- to low-resolution subnetworks successively to form a stage. The multi-resolution subnetworks are connected in parallel to form a total of four stages. The second, third, and fourth stages are set up by repeating the modularized multi-resolution blocks. The exchange unit, as illustrated in Fig. 5, aggregates the information of each resolution from other subnetworks^32^. Strided $3\times 3$ convolutions with stride 2 and a simple nearest neighbor sampling are applied for downsampling and upsampling, respectively. The last exchange unit outputs a feature map where the low-resolution representation is upsampled and concatenated into a high-resolution representation. The heatmap is regressed from the high-resolution representation. A previous study^31^ reported that the performance is enhanced by employing all resolution representations in comparison to solely using a high-resolution representation.

The ground truth heatmaps are defined as 2D gaussian functions with standard deviation of $\sigma$ centered on the ground truth location of $\textbf{L}_i^G$:

1 $$\begin{aligned} h_i^G (\textbf{x};\sigma ) = \frac{1}{2\pi \sigma ^2}\exp {\biggl (-\frac{\Vert \textbf{x} - \textbf{L}_i^G\Vert _2^2}{2\sigma ^2}\biggl )}, \end{aligned}$$

where, $\textbf{x}$, $i=\{1,... N\}$, and $N(=23)$ represent the heatmap pixel, index of the landmarks, and total number of landmarks, respectively. To converge the estimated landmarks as close as possible to the ground truth landmarks, the loss function employs the $L_{2}$ loss as follows:

2 $$\begin{aligned} L_c = \sum _{k=1}^N \Vert h_i^G (\textbf{x};\sigma ) - h_i^P (\textbf{x};\textbf{w})\Vert _2^2, \end{aligned}$$

where $h_i^P (\textbf{x};\textbf{w})$ represents the estimated heatmap, and $\textbf{w}$ denotes the weights and biases of the network. The loss function allows the network to learn the relationship between the local features and landmarks. The heatmap comprises a 2D matrix with 23 channels corresponding to the number of landmarks. The estimated coordinates $\textbf{L}_i^P$ of each landmark are predicted by transforming from the reduced space to the original image space. We adjusted the offset of the largest value position from the largest value to the second largest value^36^. Accordingly, the estimated position can be expressed as a set of 2D coordinates $\{\textbf{x}^P_i, \textbf{y}^P_i\}_{i=1}^N$.

### Coordinate regression

MLP is a refinement of coordinates estimated using spatial relationships between landmarks. It has a simple three-layer structure with an input layer, a hidden layer, and an output layer. Because this is a simple regression task, we adopted a simple MLP to reduce the possibility of an overfitting. In^2^, a linear filter was also employed to refine the landmark positions; however, only some landmarks were adjusted, and the inputs were the combination of outputs from the two models when considering spatial information. The estimated coordinates $\{\textbf{x}^P_i, \textbf{y}^P_i\}_{i=1}^N$ are divided into $\{\textbf{x}^P_1,...,\textbf{x}^P_N\}$ and $\{\textbf{y}^P_1,... ,\textbf{y}^P_N\}$. The *x*- and *y*-coordinates are input into separate models. We adopted the $L_{2}$ loss as the loss function to get closer to the ground truth landmarks by using the positional relationship of the landmarks:

3 $$\begin{aligned} L^x_n&= \sum _{k=1}^N \Vert \textbf{x}^G_i - \textbf{x}^R_i\Vert _2^2, \end{aligned}$$

4 $$\begin{aligned} L^x_n&= \sum _{k=1}^N \Vert \textbf{y}^G_i - \textbf{y}^R_i\Vert _2^2, \end{aligned}$$

where $\textbf{x}^G_i$ and $\textbf{y}^G_i$ represent the ground truth coordinates, and $\textbf{x}^R_i$ and $\textbf{y}^R_i$ denote the refined coordinates. Losses in the MLP were not propagated to HRNetV2, and were learned independently. The number of training epochs for HRNetV2 and MLP differed. For each epoch used for training HRNetV2, we trained the MLP with multiple epochs, provided that we initialized the parameters only at the beginning of training. This procedure allowed us to train the entire model efficiently, while fine-tuning the MLP to match the training phase of HRNetV2.

### Implementation details

We trained and carried out testing using a GeForce GTX 1080, 3.70-GHz Intel(R) Core(TM) i7-8700K CPU, and 16GB of memory. The training and testing were conducted in Pytorch. The input images were cropped and resized to $256\times 256$, according to the center positions of the boxes.

The HRNetV2 network starts with a stem comprising two strided $3\times 3$ convolutions, which reduces the resolution to 1/4. As the inputs pass through the four subsequent stages, the resolution is gradually reduced by half, and the number of channels is accordingly doubled. The first stage contains four residual units, each of which was formed by a bottleneck with a width of 64. This stage is followed by a $3\times 3$ convolution, which reduces the width of the feature map to 18. Thus, the number of channels for the four resolutions is 18, 36, 72, and 144, respectively. The second, third, and fourth stages contain one, four, and three multi-resolution blocks, respectively. One multi-resolution block contains four residual units. Each unit contains two $3\times 3$ convolutions for each resolution and an exchange unit across resolutions. The four resolution representations from the fourth stage are concatenated and used to predict heatmaps with a width of 64, following two $1\times 1$ convolutions. We trained HRNetV2 with 60 epochs and a batch size of 16. The model was pre-trained using WFLW^37^. The base learning rate was set as 0.0001, and then was reduced to 0.00001 and 0.000001 at 30 and 50 epochs, respectively. The loss function was minimized using the Adam optimizer.

The MLP network comprises three layers: input, hidden, and output layers. The number of neurons in the middle layer was set to 500. The number of inputs and outputs was set to 23 for splitting the coordinates transformed from the heatmaps and predicted by HRNetV2 into *x*- and *y*-coordinates. We trained the MLP using 100 epochs and a batch size of 16 every time HRNetV2 was trained for 1 epoch. The loss function was minimized using the Adam optimizer with a learning rate of 0.00001 and a weight decay (L2 regularization) factor of 0.0001.

### Evaluation metrics

We evaluated the proposed method in terms of the mean radial error (MRE), successful detection rate (SDR), and successful classification rate (SCR), according to a previous benchmark study^13^. The radius error was defined by $R=\sqrt{\varDelta x^2+\varDelta y^2}$, where $\varDelta x$ and $\varDelta y$ represent the Euclidean distances between the estimated landmarks and the ground truth landmarks of the *x*- and *y*-axes, respectively. The MRE and the standard deviation (SD) are defined as follows:

5 $$\begin{aligned} \textrm{MRE}&= \frac{\sum _{i=1}^N R_i}{n}, \end{aligned}$$

6 $$\begin{aligned} \textrm{SD}&= \sqrt{\frac{\sum _{i=1}^N (R_i-MRE)^2}{n-1}}, \end{aligned}$$

where $R_i$ represents the radial error of the *i*th landmark, and *n* denotes the total number of landmarks to be detected. The SDR is the ratio of estimated landmarks that are within a reference threshold, and is defined as follows:

7 $$\begin{aligned} SDR = \frac{n_d}{n}\times 100\%, \end{aligned}$$

where $n_d$ represents the number of successfully detected landmarks, and the threshold values are 2.0, 2.5, 3.0, and 4.0 mm, as typically used. The SCR is the classification accuracy of anatomical face types based on eight clinical measures (ANB, SNB, SNA, overbite depth indicator (ODI), anteroposterior dysplasia indicator (APDI), facial height index (FHI), frankfurt mandibular angle (FMA), modified wits (MW)). Facial images are classified into three anatomical types under clinical measures. Note that geometric criteria such as the angles and distances between landmarks listed in Table 1 are considered.

**Table 1:** Criteria for eight clinical measures of anatomical face-type classification in SCR.

Method	type1	type2	type3
$\text {ANB}$	Class 1 (normal): $3.2^\circ$ – $5.7^\circ$	Class 2: $> 5.7^\circ$	Class 3: $< 3.2^\circ$
$\text {SNB}$	Normal mandible: $74.6^\circ$ – $78.7^\circ$	Retrognathic mandible: $> 74.6^\circ$	Prognathic mandible: $< 78.7^\circ$
$\text {SNA}$	Normal mandible: $79.4^\circ$ – $83.2^\circ$	Prognathic maxilla: $> 83.2^\circ$	Retrognathic maxilla: $< 79.4^\circ$
$\text {ODI}$	Normal: $78.4^\circ$ – $80.5^\circ$	Deep bite tendency: $> 80.5^\circ$	Open tendency: $< 68.4^\circ$
$\text {APDI}$	Normal: $77.6^\circ$ – $85.2^\circ$	Class 2 tendency: $< 77.6^\circ$	Class 3 tendency: $> 85.2^\circ$
$\text {FHI}$	Normal: 0.65 – 0.75	Short face tendency: $> 0.75$	Long face tendency: $< 0.65$
$\text {FMA}$	Normal: $26.8^\circ$ – $31.4^\circ$	Mandible high angle tendency: $> 31.4^\circ$	Mandible lower angle tendency: $< 26.8^\circ$
$\text {MW}$	Normal: 2 – $4.5 \text {mm}$	Edge to edge: 0 mm	Large over jet: $> 4.5$ mm

ANB is the angle between L5, L2, and L6. SNB is the angle between L1, L2, and L6. SNA is the angle between L1, L2, and L5. ODI is the arithmetic sum of the angle between the lines from L5 to L6 and from L8 to L10, and the angle between the lines from L3 to L4 and from L17 to L18. APDI is the arithmetic sum of the angle between the lines from L3 to L4 and from L2 to L7, the angle between the lines from L2 to L7 and from L5 to L6, and the angle between the lines from L3 to L4 and from L17 to L18. FHI is the ratio of the distance from L1 to L10 (PFH) to the distance from L2 to L8 (AFH). FMA is the angle between the line from L1 to L2 and the line from L10 to L9. MW is the distance between L12 and L11.

## Results

### Accuracy and reliability of the superimposition of cephalometric tracing on the facial profile image

All distance errors were considered to follow a normal distribution by the Shapiro-Wilk test (p>0.05; not shown for details). The mean landmark distance error, standard deviation and the ICC values for each set are shown in Table 2. The mean intra- and inter-observer landmark distance error and standard deviations were 0.32 mm ± 0.07 mm and 0.41 mm ± 0.11 mm, respectively, indicating superiority over previous studies^18^. The ICC of the mean landmark distance error for each patient ranged from 0.993 to 0.998 (< 0.00, poor; 0.00–0.20, slight; 0.21–0.40, fair; 0.41–0.60, moderate; 0.61–0.80, substantial; 0.81–1.00, almost perfect)^38^, indicating a high degree of intra- and inter-observer agreement. The 95% confidence interval also supports intra- and inter-observer agreement. In this way, the accuracy and reliability of the superposition was confirmed.

**Table 2:** Intra- and inter-observer mean landmark distance error, standard deviation and the ICC values with 95% confidence intervals for five sets.

Set	The intra-observer landmark distance error (mm)	The inter-observer landmark distance error (mm)	ICC(1, 3) with 95% confidence intervals	ICC(2, 3) with 95% confidence intervals
1	0.37 ± 0.06	0.27 ± 0.04	0.996 (0.984–1.000)	0.993 (0.968–0.999)
2	0.32 ± 0.06	0.41 ± 0.10	0.996 (0.983–1.000)	0.998 (0.992–1.000)
3	0.32 ± 0.07	0.41 ± 0.09	0.996 (0.982–1.000)	0.998 (0.987–1.000)
4	0.32 ± 0.07	0.46 ± 0.10	0.996 (0.983–1.000)	0.998 (0.991–1.000)
5	0.29 ± 0.07	0.49 ± 0.07	0.997 (0.973–0.999)	0.996 (0.980–0.999)

### Result of repeatability and reproducibility test of manual landmark plotting

All distance errors were considered to follow a normal distribution by the Shapiro-Wilk test (p>0.05; not shown for details). The mean prediction error, standard deviation and the ICC values for each landmark are shown in Table 3. The mean intra- and inter-observer prediction error and standard deviations were 0.38 mm ± 0.11 mm and 0.37 mm ± 0.11 mm, respectively, indicating superiority over previous studies^18^. The ICC of the prediction error for each landmark ranged from 0.992 to 0.999 (< 0.00, poor; 0.00–0.20, slight; 0.21–0.40, fair; 0.41–0.60, moderate; 0.61–0.80, substantial; 0.81–1.00, almost perfect)^38^, indicating a high degree of intra- and inter-observer agreement. The 95% confidence interval also supports intra- and inter-observer agreement. In this way, intra- and inter-observer agreement was confirmed.

**Table 3:** Intra- and inter-observer mean prediction error, standard deviation and the ICC values with 95% confidence intervals for 23 landmarks.

Landmark	The intra-observer prediction error (mm)	The inter-observer prediction error (mm)	ICC(1, 3) with 95% confidence intervals	ICC(2, 3) with 95% confidence intervals
L1	0.32 ± 0.06	0.39 ± 0.08	0.994 (0.971–0.999)	0.993 (0.970–0.999)
L2	0.34 ± 0.06	0.40 ± 0.07	0.996 (0.981–1.000)	0.995 (0.979–0.999)
L3	0.36 ± 0.08	0.40 ± 0.11	0.997 (0.985–1.000)	0.993 (0.968–0.999)
L4	0.38 ± 0.06	0.39 ± 0.09	0.993 (0.968–0.999)	0.994 (0.975–0.999)
L5	0.36 ± 0.10	0.43 ± 0.10	0.996 (0.982–1.000)	0.992 (0.964–0.999)
L6	0.35 ± 0.10	0.46 ± 0.09	0.998 (0.991–1.000)	0.993 (0.966–0.999)
L7	0.41 ± 0.09	0.48 ± 0.05	0.997 (0.987–1.000)	0.994 (0.975–0.999)
L8	0.44 ± 0.07	0.44 ± 0.10	0.994 (0.972–0.999)	0.993 (0.965–0.999)
L9	0.42 ± 0.08	0.45 ± 0.06	0.995 (0.979–0.999)	0.996 (0.979–1.000)
L10	0.39 ± 0.10	0.33 ± 0.07	0.993 (0.971–0.999)	0.993 (0.969–0.999)
L11	0.25 ± 0.05	0.22 ± 0.05	0.992 (0.965–0.999)	0.994 (0.967–0.999)
L12	0.25 ± 0.06	0.24 ± 0.04	0.994 (0.971–0.999)	0.993 (0.969–0.999)
L13	0.46 ± 0.08	0.30 ± 0.04	0.993 (0.967–0.999)	0.993 (0.967–0.999)
L14	0.52 ± 0.06	0.35 ± 0.06	0.993 (0.967–0.999)	0.998 (0.992–1.000)
L15	0.51 ± 0.06	0.33 ± 0.08	0.995 (0.975–0.999)	0.998 (0.981–1.000)
L16	0.55 ± 0.06	0.39 ± 0.10	0.994 (0.974–0.999)	0.997 (0.984–1.000)
L17	0.34 ± 0.05	0.29 ± 0.07	0.994 (0.972–0.999)	0.996 (0.981–1.000)
L18	0.37 ± 0.07	0.29 ± 0.07	0.993 (0.968–0.999)	0.997 (0.986–1.000)
L19	0.28 ± 0.06	0.33 ± 0.09	0.993 (0.968–0.999)	0.998 (0.991–1.000)
L20	0.46 ± 0.06	0.43 ± 0.11	0.994 (0.973–0.999)	0.999 (0.994–1.000)
L21	0.47 ± 0.06	0.46 ± 0.08	0.995 (0.977–0.999)	0.998 (0.989–1.000)
L22	0.23 ± 0.05	0.46 ± 0.07	0.992 (0.966–0.999)	0.997 (0.985–1.000)
L23	0.22 ± 0.06	0.35 ± 0.08	0.996 (0.981–1.000)	0.996 (0.990–1.000)

### Performance of cephalometric landmark detection for the proposed model and comparison with existing methods

**Figure 6:**
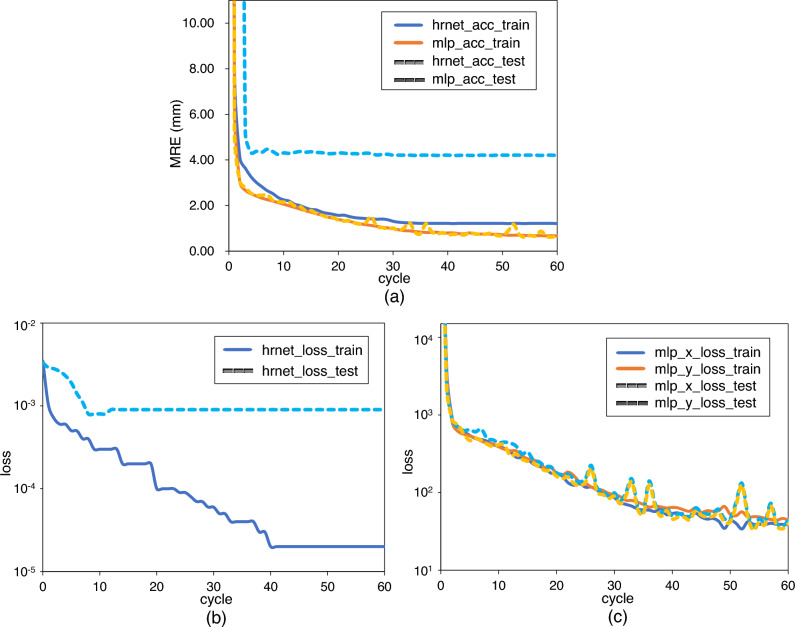
Performances of deep convolutional neural network-based AI model. (**a**) MRE of training and test according to the number of cycles. (**b**) HRNetV2 Loss of training and test according to the number of cycles. (**c**) MLP Loss of training and test according to the number of cycles. Cycle means the process of training one epoch of HRNetV2 followed by 100 epochs of MLP.

Table 4 presents a comparison between the results of the proposed and existing methods. The proposed method achieves the best performance under all metrics. Because no existing studies have solely adopted facial profile images for training, we provide comparisons with a study adopting X-rays. The comparison methods were selected based on their recency and proximity to the proposed method. The performance of the proposed model trained on facial profile images is presented in Table 7. Table 5 presents the MRE, SD, and SDR for each of the 23 landmarks. The MRE for all landmarks is less than 2 mm, which is clinically acceptable^6^. Among the landmarks, Basion exhibited the best MRE. However, the MRE of Sella and Mo are large, and the SDR at 2 mm is low. The results of the proposed method indicates that Orbitale, Subspinale, Pogonion, Gonion, and Articulare, which have been known to handle landmarks that are difficult to accurately estimate in previous studies^7,8,13,19^, can be estimated within 1 mm. In addition, it can be seen that the error in the *y*-coordinate is larger than that in the *x*-coordinate. Table 6 presents the SCR of the proposed method and the existing approachs. In terms of classification, the proposed method outperforms the existing methods under all metrics. The classification accuracy exceeds 90% in six out of eight metrics. Fig. 6 shows the MRE and loss of training and test according to the number of cycles. We defined one cycle as the process of training one epoch of HRNetV2 followed by 100 epochs of MLP.

**Table 4:** Comparison of MRE and SDR for the automated cephalometric analysis systems.

Method	MRE (mm)	SDR (%)			
		2.0 mm	2.5 mm	3.0 mm	4.0 mm
Ibragimov et al.^14^	1.96	68.13	74.63	79.77	86.87
Lindner et al.^15^	1.77	71.65	76.93	82.17	89.85
Arik et al.^18^	-	72.30	78.21	82.24	86.81
Gilmour et al.^9^	1.14	83.81	89.14	93.22	97.13
Li et al.^10^	1.20	83.72	89.34	92.72	96.78
Kwon et al.^2^	1.24	83.01	88.78	92.21	96.59
Oh et al.^19^	1.29	82.08	88.06	92.34	96.92
Proposed	**0.85**	**96.35**	**98.80**	**99.64**	**99.99**

All methods were trained by the ISBI2015 dataset^13^. The averages using test1 and test2 are reported. Significant values are in bold.

### Ablation study

**Figure 7:**
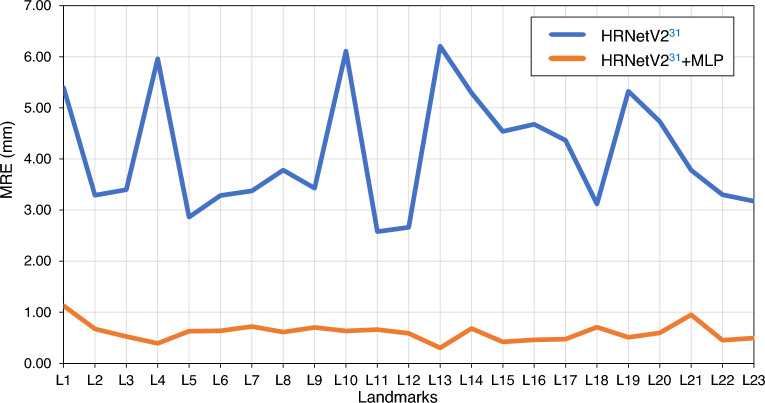
MRE for each landmark, with and without MLP.

**Figure 8:**
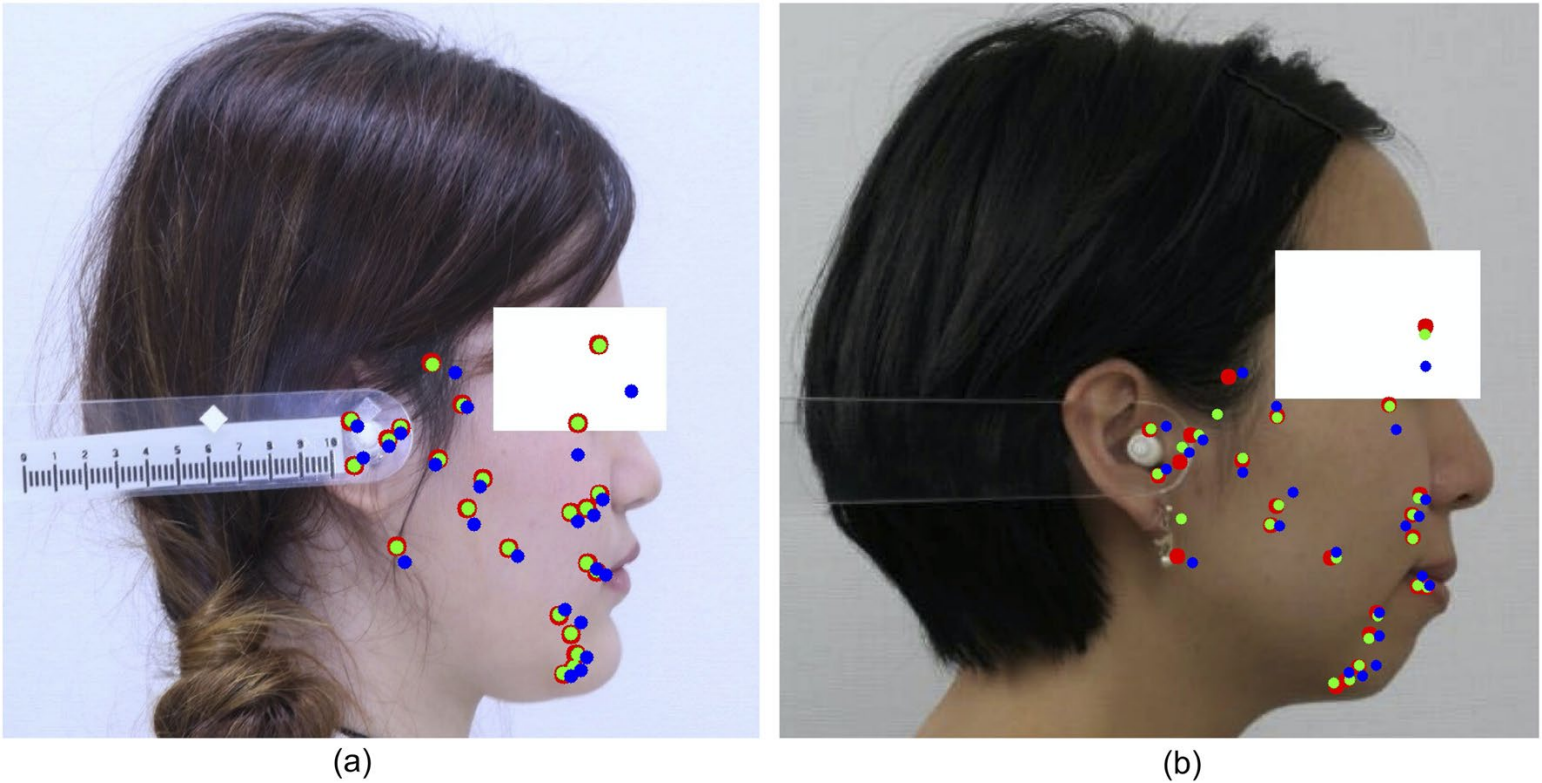
Visualization of landmarks, with and without MLP. (**a,b**) The best and worst result of the model with MLP, respectively. Blue and green dots show the estimations by HRNetV2 and HRNetV2+MLP, respectively, and the red dots indicate the ground truth.

We evaluated the contribution of MLP to the proposed method. We compared the performance of HRNetV2^31^ with the performance of HRNetV2 combined with MLP. Table 7 presents the MRE, SD, and SDR for each model. Accordingly, it was deduced that the application of MLP significantly improves the performance. This indicates that heatmap regression, followed by coordinate regression using MLP, works effectively. The MRE for each landmark is presented in Fig. 7. The accuracies for Sella, Porion, Gonion, Basion, and Articulare, where HRNetV2 exhibits a poor accuracy, were also significantly improved. Table 8 presents the SCR for each. From this Table, it is evident that the accuracy is significantly improved, except for MW, which is consistently high. Figure 8 presents a visualization of the estimations by HRNetV2 and HRNetV2 + MLP, including the ground truth locations of the landmarks.

**Table 5:** MRE, SD, and SDR for each landmark.

Landmark	MRE (mm)	SD (mm)	*x*-direction		*y*-direction		SDR (%)			
			$\varDelta x$ (mm)	SD (mm)	$\varDelta y$ (mm)	SD (mm)	2.0 mm	2.5 mm	3.0 mm	4.0 mm
L1	1.13	1.39	0.44	0.31	0.91	1.37	89.95	95.35	97.50	99.25
L2	0.67	0.33	0.28	0.19	0.56	0.35	99.70	99.90	99.95	100
L3	0.53	0.30	0.26	0.19	0.40	0.30	99.80	99.90	100	100
L4	0.39	0.16	0.30	0.16	0.20	0.13	100	100	100	100
L5	0.63	0.36	0.43	0.35	0.37	0.29	99.40	99.80	99.95	99.95
L6	0.64	0.35	0.32	0.25	0.48	0.34	99.50	99.95	99.95	99.95
L7	0.72	0.41	0.38	0.29	0.53	0.42	99.00	99.70	99.95	100
L8	0.61	0.37	0.36	0.27	0.41	0.35	99.45	99.85	99.90	99.95
L9	0.70	0.40	0.41	0.31	0.48	0.31	98.90	99.80	99.90	100
L10	0.63	0.69	0.19	0.14	0.56	0.71	98.85	99.65	99.80	99.90
L11	0.66	0.37	0.43	0.34	0.41	0.31	99.45	99.70	99.90	99.95
L12	0.59	0.36	0.37	0.31	0.38	0.31	99.80	99.95	99.95	99.95
L13	0.30	0.15	0.17	0.13	0.21	0.14	100	100	100	100
L14	0.68	0.54	0.45	0.35	0.41	0.51	99.50	99.85	99.90	99.90
L15	0.42	0.14	0.23	0.14	0.31	0.14	100	100	100	100
L16	0.46	0.28	0.36	0.28	0.22	0.18	99.90	99.90	99.90	99.95
L17	0.47	0.20	0.33	0.18	0.27	0.18	100	100	100	100
L18	0.71	0.39	0.52	0.39	0.38	0.30	98.60	99.75	99.90	99.95
L19	0.51	0.29	0.37	0.29	0.28	0.20	99.90	99.95	99.95	100
L20	0.60	0.29	0.39	0.26	0.37	0.26	99.85	100	100	100
L21	0.95	0.62	0.45	0.36	0.75	0.63	92.45	96.95	99.00	99.85
L22	0.45	0.25	0.28	0.22	0.30	0.23	99.80	99.95	100	100
L23	0.50	0.29	0.37	0.25	0.27	0.24	99.35	99.75	99.80	99.95
Average	0.61	0.53	0.35	0.30	0.41	0.51	98.83	99.55	99.79	99.93

**Table 6:** Comparison of SCR for the automated cephalometric analysis systems. Numbers are given in percentages (%).

Method	ANB	SNB	SNA	ODI	APDI	FHI	FMA	MW
Ibragimov et al.^14^	66.31	72.75	63.50	72.31	80.07	70.08	77.78	81.38
Lindner et al.^15^	69.33	83.48	72.26	79.29	84.18	77.11	77.59	82.16
Arik et al.^18^	67.81	69.99	64.83	73.94	84.30	67.22	75.54	79.77
Kwon et al.^2^	81.78	84.14	72.91	85.26	87.47	86.40	85.75	87.50
Oh et al.^19^	80.90	85.23	68.98	79.01	86.15	82.48	80.98	87.73
Proposed	**90.45**	**87.30**	**83.50**	**93.75**	**91.50**	**93.55**	**93.55**	**98.65**

Significant values are in bold.

**Table 7:** Performance of the proposed method and comparison of MRE, SD, and SDR, with and without MLP.

Method	MRE (mm)	SD (mm)	SDR (%)			
			2.0 mm	2.5 mm	3.0 mm	4.0 mm
HRNetV2^31^	4.11	3.21	22.42	32.23	40.74	58.20
HRNetV2^31^+MLP	0.61	0.53	98.20	99.55	99.79	99.93

**Table 8:** SCR comparison, with and without MLP.

Method	ANB	SNB	SNA	ODI	APDI	FHI	FMA	MW
HRNetV2^31^	66.30	56.75	44.60	65.70	64.00	76.70	79.35	97.65
HRNetV2^31^+MLP	90.45	87.30	83.50	93.75	91.50	93.55	93.55	98.65

## Discussion

The proposed method is novel because it estimates the location of cephalometric landmarks without lateral cephalograms. Table 7 shows that the MRE of the proposed method is 0.61 mm. While a direct comparison is difficult due to differences in datasets, the proposed method demonstrates higher performance compared to the previously reported error^18^ between expert clinicians and existing methods. The experimental results suggest that the proposed method may perform at a level equal to or potentially better than existing methods using cephalograms. Although a manual analysis is limited by inter- and intra-person errors, the proposed model achieves an SD of 1 mm for all landmarks except for Sella. This implies that the proposed model can provide a stable estimation with small deviations. Furthermore, the proposed method achieves a significant SDR and is free from the effects of extreme outliers^18,19^ which have limited several previous studies. Therefore, a cephalometric analysis using AI may replace a human analysis in the future.

The accuracy is also high for landmarks that have been considered difficult to estimate accurately in existing studies. Figure 7 indicates that the estimations of Gonion and Articulare by HRNetV2 are still inaccurate. This may be due to the influence of the ear rod used to fix the head position, which is commonly included in both lateral cephalograms and facial profile images. This supports the idea that CNN-based heatmap regression methods learn each landmark independently. In particular, HRNetV2 strictly learns the positional relationship of each landmark with the surrounding features owing to the parallel distributed processing with many convolutional layers applied. Following the estimation with HRNetV2, the refinement of the landmark locations using MLP contributes to an improvement of the estimation accuracy of all landmarks. It is difficult to learn intricate positional relationships using only MLP. Note that the estimation in HRnetV2 described in the previous section may include the potential structural relationships among landmarks. Because MLP is fully connected from the input layer to the output layer and the input location information is processed comprehensively, it is possible to explicitly learn this potential positional relationship. This should allow MLP to incorporate the structural features among landmarks into the estimation, which should lead to a significant improvement in accuracy. The effectiveness of incorporating structural relationships among landmarks in an estimation was also shown by Oh et al.^19^. Although Fig. 6a, b show that HRNetV2, which provides the intermediate output, tends to overfit, Fig. 6a, c show that the loss of training and test data is very similar in the MLP that provides the final output, indicating almost no over-fitting issue. This suggests that MLP is effective in reducing the effects of over-fitting by HRNetV2. Increasing the number of data may reduce over-fitting in HRNetV2 and further improve overall accuracy. In addition, enhancing the heatmap regression procedure can further enhance the overall performance. We can introduce adaptive wing loss^34^ or face boundary prediction^33^, which achieves a high accuracy in facial landmark detection.

As demonstrated in Table 5, Sella, and Mo have a larger MRE and lower SDR at 2 mm than the other landmarks. In particular, Sella has a larger error in the *y*-direction. Two reasons can be given for the estimation difficulty: First, Sella is the highest position among the landmarks to be estimated in most cases, and its location is far from the jaw area where the landmarks are densely located. Second, Mo has large errors in both the *x*- and *y*-directions. It appears that the variation of the position among patients biased the model. The error tends to be larger in the *y*-direction than in the *x*-direction. The shape of the face may influence the estimation. Most of the patients in this study have long faces, and landmarks are scattered in the *y*-direction. This may explain why the estimation error in the *y*-direction is larger than that in the *x*-direction.

Table 6 implies that the proposed method achieves a significant SCR. Because the SCR is calculated using linear and angular measurements, it is not influenced by the pixel spacing. Therefore, we can make reliable comparisons, even with different datasets. Note that the dataset used in this study did not include patients with a malocclusion. However, a clinical evaluation requires the inclusion of such data.

The performance of the proposed method tends to depend on the size and diversity of the training data. Previous studies^39^ have reported that the accuracy of the proposed method increases with an increase in the number of data. Hence, adding more data is the easiest way to improve the system. However, this study also includes the risk of bias regarding the validation discussed in Schwendicke et al.^40^. Because the MLP approach is model-agnostic, there is no restriction on the heatmap regression model used for the initial estimation. The proposed method can reconcile the local features of images with the spatial relationship between landmarks. Hence, it may be widely used for landmark detection tasks such as facial landmark detection and pose estimation. In the future, it will be necessary to address 3D landmark detection.

## Conclusion

In this paper, we proposed a novel cephalometric landmark detection method without the use of X-rays. The proposed framework combines the CNN-based heatmap regression model (HRNetV2) with a coordinate regression model (MLP). HRNetV2 estimates the location of landmarks by learning the relationship between local features and landmarks. However, it is limited by the same problem as conventional heatmap regression methods, and its accuracy is insufficient. The MLP following HRNetV2 can learn the spatial positional relationship between landmarks, which significantly improves the accuracy of the estimation. In experiments conducted with the created dataset, the proposed method performed remarkably with an MRE of 0.61 mm and a detection rate within 2.0 mm of 98.20%. The proposed method also achieved a high accuracy in terms of anatomical face-type classification, where a reliable comparison between different datasets is possible. In the future, in addition to replacing existing methods using X-rays, the proposed method may also replace measurements by humans. Because the proposed landmark refinement using MLP is model-agnostic, it can be combined with conventional methods. Furthermore, it has the potential to be a prominent approach applicable to various landmark detection tasks and provide significant improvements in performance.

## Data Availability

The datasets generated and analyzed during the study are not publicly available because sensitive information in them may violate patient privacy and our institution’s ethics policy. However, the datasets are available from the corresponding author on reasonable request. Data usage agreements may be required.
